# Evaluation of pharmacist’s interventions in a Hospital Pharmacy of a Secondary Care Centre of Aga Khan University Hospital

**DOI:** 10.12669/pjms.41.9.11700

**Published:** 2025-09

**Authors:** Afia Tariq Butt, Ayesha Feeroze, Iyad Naeem, Sumaira Khadim

**Affiliations:** 1 Afia Tariq Butt, FCPS Senior Instructor, Department of Pediatric Medicine and Child Health, Aga Khan University Hospital, Karachi, Pakistan; 2 Ayesha Feeroze, Pharm-D Department of Pharmacy, Aga Khan Hospital for Women, Karachi, Pakistan; 3 Iyad Naeem, Ph.D Professor, Head of Department, Pharmaceutics, University of Karachi, Pakistan; 4 Sumaira Khadim, Ph.D Faculty of Pharmacy, Iqra University, Karachi, Pakistan

**Keywords:** Prescribing error, Intervention, Secondary care

## Abstract

**Objective:**

The current study aimed to investigate pharmacists’ intervention statistics in a secondary care setting in Pakistan.

**Methodology:**

A retrospective cross-sectional study was conducted at a secondary care facility of Aga Khan University, Karachi, Pakistan, analyzing in-patient and out-patient pharmacy records from June 2022 to June 2023.

**Results:**

The study analyzed records of 10.9% (n=1340) male and female patients with a median age of six years (IQR: 23 years). Pharmacists intervened in 99.1% (n=1329) of prescriptions due to prescribing errors (PEs). The most common intervention was therapy optimization (e.g., therapeutic drug monitoring, incomplete prescriptions, wrong doses/frequencies, updating drug allergies, following culture/sensitivity results and monitoring electrolytes), accounting for 72.4% (n=970) of interventions, followed by route conversion at 19.6% (n=263). The overall acceptance rate of pharmacists’ interventions among physicians was 88.7% (n=1189).

**Conclusion:**

PEs were common and pharmacists’ interventions were vital in preventing patient harm. PEs were more frequent among in-patients at risk of polypharmacy. Pharmacists optimized therapy across all age groups by adjusting doses, administration routes and applying restrictions. The study noted high acceptance of these interventions.

## INTRODUCTION

The role of pharmacists has expanded from dispensing medications to patient care, counseling, health education, community service and clinical practice. The World Health Organization estimates that over half of prescribed drugs are dispensed or used inappropriately and about half of patients misuse them.^1^ Drug-related problems (DRPs) can lead to adverse effects, drug interactions, resistance and increased medication costs.^2^ A DRP is defined as “an event or circumstance involving drug therapy that actually or potentially interferes with desired health outcomes,” often recognized as “Prescribing Errors” (PEs).^3^ Pharmacist interventions are crucial in optimizing drug therapy and preventing DRPs. Documenting these interventions is essential to justify their importance to healthcare administrators, providers and caregivers. Interventions range from handwritten forms to computerized databases.^4^

Globally, pharmacists’ contributions are recognized, but in underdeveloped countries like Pakistan, the situation is dire, with few skilled pharmacists, as many opt for the industry sector over hospitals.^4^ Limited resources in Pakistan’s healthcare system hinder medication error reporting and documentation, often relying on manual or semi-electronic records. Pharmacist-led interventions reduced DRPs from 88.8% to 74.9% in Vietnam.^2^ In Saudi Arabia’s Jazan Region, 369 interventions were recorded over two years.^5^ A German University Hospital study showed that a pharmacist intervened in every fifth medication review.^6^

In a tertiary hospital in Hyderabad, Pakistan, 1.41 medication errors per prescription were observed.^7^ Secondary care hospitals, serving diverse populations, play a vital role in easing healthcare system pressure. Pharmacist interventions improve prescribing safety, integrated care, patient satisfaction and provider job satisfaction^8^ while numerous studies have examined pharmacist interventions in community and tertiary hospitals, none have focused on secondary care hospitals in underdeveloped regions like Pakistan. This study aimed to analyze pharmacist intervention statistics in a secondary care setting, estimating the types and frequency of prescribing errors and pharmacist interventions and assessing physicians’ acceptance. It also explores differences in interventions between in-patient and out-patient settings.

## METHODOLOGY

A retrospective cross-sectional study was conducted at Aga Khan Hospital, Garden, Karachi, Pakistan, analyzing pharmacy service records for in-patients and out-patients from June 2022 to June 2023. The hospital used an electronic system where pharmacists logged interventions in the pharmacy software during prescription reviews for out-patients or physician-entered orders for in-patients. Interventions were recorded under a unique “Rx” number linked to the patient profile, capturing details such as prescribing errors, intervention type, significance, physician identification and acceptance status to ensure patient safety and quality care.

A prescribing error (PE) was defined as a clinically substantial error resulting from prescribing decisions or processes that reduce the treatment effectiveness or timeliness.^9^ PEs were categorized as: (i) wrong dose, (ii) wrong medication, (iii) duplicate therapy, (iv) contraindicated drugs, (v) medication prescribed despite documented ADR/allergy, (vi) wrong administration time, (vii) wrong route, (viii) wrong frequency, (ix) incomplete prescription and (x) others.^9^ A pharmacy intervention was defined as any proposed measure to be taken by the pharmacist to overcome the potential problem related to a medical prescription or a medication order to prevent or solve the problem 9 .Interventions were classified into 12 types: (i) updating drug allergy status, (ii) stopping contraindicated medications, (iii) pharmacotherapeutic recommendations, (iv) preventing pharmaceutical incompatibility, (v) IV-to-oral conversions, (vi) discontinuing medications without indication, (vii) optimizing therapy monitoring (i.e. therapeutic drug monitoring, prescriptions information quality or incomplete prescription, correcting wrong dose and wrong frequency, updating drug allergy, following culture/sensitivity results, clear instructions given regarding electrolytes monitoring), (viii) dose adjustments for hepatic/renal conditions, (ix) approving restricted medications, (x) preventing therapeutic duplications, (xi) recommending treatment for untreated indications and (xii) other unclassified actions.^9^ The goal was to enhance patients` health and well-being, with the study also assessing physicians’ acceptance of interventions. PEs were further categorized by the potential for harm: insignificant (no permanent harm but requiring monitoring), significant (potential temporary harm) and highly significant (potential for hospitalization, permanent damage, near-death, or death).^10^

### Data collection procedures:

A structured proforma was designed to extract required information from the pharmacy’s electronic database, including patient demographics (age and sex), frequency and type of PEs, pharmacists’ interventions and their acceptance by the physicians.

### Data analysis:

Data was entered into SPSS. Descriptive statistics were calculated for patients` characteristics. Median and interquartile range (IQR) were used for continuous variables, while frequencies were calculated for PEs, pharmacists’ interventions and physician acceptance. The Chi-square test was applied to identify significant differences in PEs and physician responses to interventions.

### Ethical considerations:

Ethical approval was obtained from the Ethics Review Committee (Reference no.2024-8795-28052; Dated: February 9, 2024) of Aga Khan University Hospital, Karachi. Confidentiality was ensured through anonymity and limited data access.

## RESULTS

The total number of prescriptions dispensed from the pharmacy at The Aga Khan Hospital, Garden, during the period from 1^st^ June 2022 to 30^th^ June 2023, was approximately 12,300**.** The current study retrieved records of total 10.9% (n=1340) male and female patients in age group ranging from children to the elderly for whom any kind of pharmacists` intervention was offered. The median age of the patients was 6 years with an IQR of 23 years. 38.4% (n=515) of all the patients were males while 61.6% (n=825) were females. Among all 1340 patients included in the analysis, 99.1% (n=1329) of patients` prescriptions were intervened by the pharmacists. While 0.9% (n=11) were intervened for pharmacists` identified therapeutic recommendations. Hence, the total frequency of PE was 10.8% (n =1329). 85.4% (n =1144) of all patients were in-patients and 14.6% (n=196) were out-patients visiting clinics for different out-patient medical consultation services. Among all the prescribing errors, the most common errors were the prescription of the wrong dose, followed by the wrong route of administration. However, prescribing the wrong medication was the least reported of all the PEs, i.e., 0.1% (n=01), while no observation was recorded for unauthorized use or prescription of any medicine and prescribing a contraindicated medication.

Among various pharmacists’ interventions, optimization of therapy monitoring was the most frequent intervention comprising 72.4% (n=970) of all the interventions followed by the conversion of drug administration route i.e. 19.6% (n=263). The study also observed a high acceptance rate of 88.7% (n=1189) for the pharmacists` interventions among the physicians (Table-I).

**Table-I:** Distribution of demographic characteristics of the patients availing pharmacy services during the study period and frequency of various prescribing errors and pharmacists’ interventions as reported by pharmacy of a secondary care private hospital in Karachi, Pakistan (n =1340).

Variable	Frequency (n)	Percentage (%)
***Median age: 6 years (IQR: 23years)***		
Age		
0-18 years	907	67.0
19-35 years	330	24.6
36-65 years	96	7.2
66 years and above	07	0.5
***Sex***		
Male	515	38.4
Female	825	61.6
***Type of patient***		
In-patient	1144	85.4
Out-patient	196	14.6
***Type of prescribing error (n=1332)***		
Prescribing wrong dose	849	63.9
Prescribing wrong medication	01	0.1
Prescribing duplicate therapy	37	2.8
Prescribing medication with ADR*	03	0.2
Prescribing wrong route	230	17.3
Prescribing wrong frequency	116	8.7
Incomplete prescription	30	2.3
Unauthorized Use	0	0
Prescribing contraindicated medication	0	0
Others.	63	4.7
***Pharmacist’s Interventions***		
Preventing ADR	03	0.2
Pharmacotherapeutic recommendation	06	0.4
Conversion of drug administration route	263	19.6
Discontinue medications with no indication	01	0.1
Optimizing therapy monitoring	970	72.4
Hepatic and renal dose adjustment	04	0.3
Therapeutic duplication	36	2.7
Others	57	4.3
***Acceptance of pharmacists ‘interventions***		
Accepted by the physician	1189	88.7
Not accepted by the physician	151	11.3

* ADR= Adverse drug reaction

Others included writing wrong mnemonic, wrong duration and wrong patient selected.

**Fig.1:**
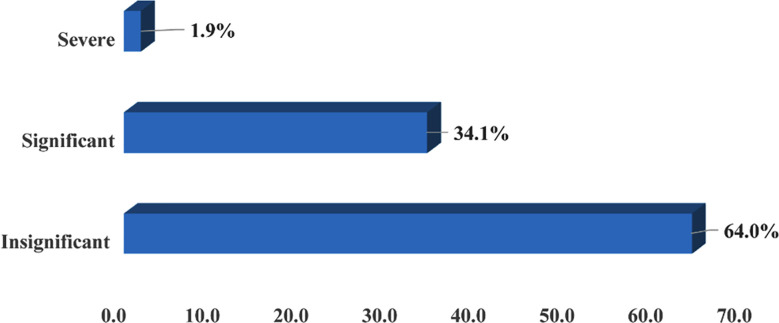
Distribution of PEs based on potential for harm (n =1340).

Only 1.9% (n =26) of all the PEs were identified as severely harmful or highly significant with potential of hospitalization or permanent damage while 34.1% (n=457) were significantly harmful with the potential of temporary harm. 64.0% (n=857) of all PEs were insignificant to cause any irreversible or considerable damage to patients` health and safety (Fig.1). Almost all kind of PEs were relatively more frequent among in-patients as compared to out-patients (Table-II).

**Table-II:** Distribution of various pharmacists’ interventions among patients receiving healthcare services at a secondary care private hospital in Karachi, Pakistan (n =1340).

Pharmacist’s interventions	In-patients n (%)	Out-patients n (%)
Preventing ADR*	1(33.3)	02(66.7)
Stopping contraindicated medications	0	0
Pharmacotherapeutic recommendation	0	06(100.0)
Preventing pharmaceutical incompatibility	0	0
Conversion of drug administration route	254(96.6)	09(3.4)
Discontinue medications with no indication	0	1(100.0)
Optimizing therapy monitoring	800(82.5)	170(17.5)
Hepatic and renal dose adjustment	04(100.0)	0
Approval of restricted medications	0	0
Therapeutic duplication	32(88.9)	04(11.1)
Recommending medications for untreated diseases	0	0
Others	53(93.0)	04(7.0)

* ADR= Adverse drug reaction

Others included writing wrong mnemonic, wrong duration and wrong patient selected.

The highest frequency of acceptance among physicians was observed for interventions related to optimizing therapy monitoring as well as Pharmacotherapeutic recommendations, i.e., 89.5%(n=868) and 83.3%(n=05) respectively. While 44.4% (n=16) of all pharmacists` interventions addressing therapeutic duplications were not accepted by the physicians (p-value <0.001) The study also found the highest acceptance rate of pharmacist interventions provided for the younger age group i.e. children as compared to higher age groups(p-value:0.001). Similarly, study found a higher acceptance rate of pharmacist intervention for male patients as compared to female patients (p-value:0.05) (Table-III).

**Table-III:** Acceptability of various pharmacists’ interventions among physicians catering patients at a secondary care private hospital in Karachi, Pakistan (n =1340).

Pharmacist’s interventions	Accepted n (%)	Not accepted n (%)	p-value
Preventing ADR	02(66.7)	01(33.3)	
Stopping contraindicated medications	0	0	<0.001
Pharmacotherapeutic recommendation	05(83.3)	01(16.7)	
Preventing pharmaceutical incompatibility	0	0	
Conversion of drug administration route	235(89.4)	28(10.6)	
Discontinue medications with no indication	01(100.0)	0	
Optimizing therapy monitoring	868(89.5)	102(10.5)	
Hepatic and renal dose adjustment	04(100.0)	0	
Approval of restricted medications	0	0	
Therapeutic duplication	20(55.6)	16(44.4)	
Recommending medications for untreated diseases	0	0	
Others	54(94.7)	03(5.3)	
***Age Groups***			
0-18 years	824(90.8)	83(9.2)	0.001
19-35 years	283(85.8)	47(14.2)	
36-60 years	77(81.1)	18(18.9)	
61 years and above	5(62.5)	3(37.5)	
***Sex***			
Male	468(90.9)	47(9.1)	0.05
Female	721(87.4)	104(12.6)	

*ADR= Adverse drug reaction

Others included writing wrong mnemonic, wrong duration and wrong patient selected.

p-value 0.05 or less is significant.

## DISCUSSION

This study examined the frequency of clinical pharmacists’ interventions and their role in ensuring medication safety for in-patients and out-patients in Pakistan. It analyzed 13 months of pharmacy data from a secondary care hospital in Karachi, covering 12,300 patients and determined a frequency of 10.9% for pharmacists` intervention, where 99.1% interventions were addressing the prescribing errors. The frequency of PEs in the current study was notably lower than similar studies reporting the frequency of PEs ranging between 35% and 60%.^9^,^11^ These differences in PE frequency can be attributed to variations in patient flow, sample size, age, facility type and in-patient vs. out-patient status. Moreover, the severity of patients presenting in secondary versus tertiary care facilities may put a different burden and have a potential to affect the prescribing errors by the physicians. Since a tertiary care caters the patients with more complicated diseases requiring complex medical treatments with more duration of hospitalization in contrast to a secondary care setup, there is more risk of PE’s.

Literature reports higher PE rates among the elderly due to polypharmacy from chronic diseases.^12^-^14^ In contrast, this study found a lower PE frequency, as most patients were children or younger, with a median age of six years. Literature reports higher PE frequencies among in-patients compared to out-patients, consistent with this study. This is due to frequent polypharmacy, medical complications and the need for careful therapy optimization and dose restrictions.^13^ Increasing the sample size or including more patients would likely reveal a higher frequency of PEs and pharmacists` interventions. The pattern of pharmacists` interventions observed in this study was well-supported by the previous evidence from multiple studies.^9^,^10^

The most frequent pharmacists` interventions in the current study was optimizing therapy monitoring, followed by conversion or change in drug administration route and preventing therapeutic duplication. This reflects a pattern similar to that observed in previous studies. However, the differences in classification of pharmacists` interventions among the studies might have slightly affected the overview, with no actual differences in the patterns.^9^,^10^,^15^ Similarly, in the current study, only 0.2% of the pharmacists’ interventions were related to Adverse Drug Reaction (ADR), which is in line with previous evidence.^9^ This low frequency for preventing ADRs can be explained by the type of medical services included in the study, i.e., non-emergency services. The likelihood of ADRs and related pharmacists’` interventions is seen higher in healthcare settings where emergency care is provided. Similarly, the frequency for preventing therapeutic duplication is also well recognized by similar studies as a common pharmacists’ intervention.^9^,^10^ Moreover, 0.4% of all interventions were pharmacotherapeutic recommendations ensuring delivery of dedicated and safe care for the patient’s well-being.

This study reported a high acceptance rate for pharmacists’ interventions in our study, with a frequency of 88.7% which is also consistent by the previous literature. However, the acceptance rate of pharmacists` interventions in our study is slightly higher than the acceptance rates reported by the previous studies in similar settings, such as Austria, Denmark, India and Turkey, with an acceptance rate for pharmacists` interventions ranging between 78% to 84%.^11^,^12^,^16^,^17^ However, the acceptance rates for pharmacists’ interventions in the current study were lower than the acceptance rates reported from studies conducted in Germany and Oman, with acceptance rates ranging from 92 to 98%.^15^,^9^ The differences in the acceptance rates for pharmacists` interventions can be explained by differences in patient population, sample size and study setting. Nevertheless, for the better acceptance of pharmacists` interventions, the role of an integrated, multidisciplinary care approach cannot be denied. Similarly, the medication review by the pharmacists is crucial for reducing Pes.^16^,^17^ The study also found significantly higher acceptance rate for pharmacists` interventions in case of a pediatric patient and if patient is a male. These findings need further exploration with large scale studies.

The study also found that most of the PEs were insignificant, with no probability of causing any irreversible damage or hospitalization or death, or near-death experiences. The current study is among the very few studies highlighting the frequency and types of prescribing errors and relevant pharmacists` interventions in a secondary care setup of an underdeveloped country like Pakistan. This study has the potential to serve as a major milestone in improving the quality of pharmacy services in Pakistan by identifying the common PEs and relevant interventions to address the existing issues particularly in a secondary care hospital of Pakistan.

Although several studies have been conducted regarding pharmacist’s intervention in tertiary care hospitals but existing research studies provide scarce data for assessing the pharmacist’s role in secondary care hospital. This study adds the crucial gap by documenting the frequency, types and physicians’ acceptance of pharmacy interventions through standardized processes in a resource-constrained secondary care setup depicting the clinical impact of pharmacy interventions on patient safety particularly in pediatric populations prone to dosing errors. The current study illustrates the highest acceptance rate of physicians (88.7%) spotlight surging of trust between the physicians and the pharmacists. The study’s strength lies in provision of real time, electronic dataset of both in-patient and out-patient pharmacy interventions that offers reliable, retrievable and consistent record. However, further study is required to explore the impact of pharmacist interventions on clinical outcomes such as duration of hospitalization and readmission rate and in evaluating the comparison of pharmacy interventions in secondary and tertiary care setups sharing the common standardized work processes. This study also opens the area for exploring the gaps of inter-professional communications and training that yield the low acceptance rate by physicians in some interventions like therapeutic duplication.

### Limitations:

This study had limitations, as its findings are not generalizable to other public or private hospitals in Karachi. It was a single-center study at a private secondary care hospital, limiting the representation of facilities with different structures or medication dispensing systems. The results are comparable only to similar settings with the automated systems and round the clock access to qualified clinical pharmacists for timely error identification and correction. Comparisons with tertiary or primary care settings may lead to underestimation or overestimation of the results. Additionally, each intervention had been done by a single pharmacist without being further reviewed by other which could lead the risk of biasness by intra-observer and pharmacist interventions were based on the judgment of one pharmacist, preventing assessment of inter-rater reliability.

## CONCLUSION

In this secondary care hospital, PEs were common and pharmacists’ interventions were vital in preventing harm. More PEs occurred among in-patients at risk of polypharmacy. Pharmacists optimized therapy across all age groups by adjusting the doses, administration routes and applying restrictions. High acceptance of these interventions has significantly reduced life-threatening and severe PEs. Enhancing pharmacist-doctor coordination must be prioritized for quality improvement and patient safety. Large multicenter studies are needed to evaluate the impact of pharmacists’ interventions on patient outcomes.

### Authors’ Contribution:

**AF and AT:** Concept, design, interpretation and analysis of data and final revision of manuscript, responsible and accountable for the accuracy and integrity of the work.

**IN and SK:** Did data collection and manuscript writing.

All authors have read and approved final version of manuscript.
